# Video Game Skills across Diverse Genres and Cognitive Functioning in Early Adulthood: Verbal and Visuospatial Short-Term and Working Memory, Hand–Eye Coordination, and Empathy

**DOI:** 10.3390/bs14100874

**Published:** 2024-09-27

**Authors:** Triantafyllia Zioga, Aristotelis Ferentinos, Eleni Konsolaki, Chrysanthi Nega, Panagiotis Kourtesis

**Affiliations:** 1 Department of Psychology, The American College of Greece, 153 42 Athens, Greeceekonsolaki@acg.edu (E.K.); cnega@acg.edu (C.N.); 2 Department of Psychology, The University of Edinburgh, Edinburgh EH8 9JZ, UK; 3 Department of Psychology, National and Kapodistrian University of Athens, 157 84 Athens, Greece

**Keywords:** video games, early adulthood, cognition, empathy, short-term memory, working memory, visuospatial memory, verbal memory, hand–eye coordination, attentional processing speed

## Abstract

The cognitive and affective impacts of video games are subjects of ongoing debate, with recent research recognizing their potential benefits. This study employs the Gaming Skill Questionnaire (GSQ) to evaluate participants’ gaming skills across six genres and overall proficiency. A total of 88 individuals aged 20–40 participated, completing assessments of empathy and six cognitive abilities: verbal short-term memory, verbal working memory, visuospatial short-term memory, visuospatial working memory, psychomotor speed (hand–eye coordination), and attention. Participants’ cognitive abilities were examined using the Digit Span Test, Corsi Block Test, and Deary–Liewald Reaction Time Task, while empathy was assessed using the Empathy Quotient Questionnaire. Findings indicate that higher levels of videogaming proficiency are linked to improvements in visuospatial short-term and working memory, psychomotor speed, and attention. Specific genres enhanced particular skills: RPGs were positively associated with both verbal working memory and visuospatial short-term memory, but were negatively associated with empathy; action games improved psychomotor speed and attention; and puzzle games showed a positive relationship with visuospatial working memory. These results add to ongoing research on the cognitive and affective effects of video games, suggesting their potential to enhance specific cognitive functions. They also highlight the complex relationship between video games and empathy. Future research should explore the long-term impacts and genre-specific effects.

## 1. Introduction

Video games have become a significant form of entertainment, comparable to the film industry in terms of profitability. In the United States, expenditure on game-related items saw a seven-fold increase from 2006 to 2023, going from a value of $7.4 billion to $57.2 billion [1,2]. This expansive industry encompasses both hardware—such as video game consoles, personal computers, smartphones, and virtual reality (VR) devices—and software in the form of the games themselves. The development of gaming culture has led to the creation of a gamer identity, characterized by choices in gaming platforms, genres, specific games, and playing styles [3,4,5]. Additionally, electronic sports have emerged (eSports), where top players compete for substantial cash prizes, highlighting the importance of understanding the effects of video games on cognitive and affective aspects in early adulthood [6,7].

Video games are designed in various genres to cater to different preferences and demands, including adventure, action, sports, role-playing (RPG), racing, strategy, puzzles, martial arts, and first-person shooters (FPS) [8]. Each genre requires different skill sets and levels of engagement, making video games attractive to a wide audience. The difficulty levels of these games are carefully balanced to maintain player interest and satisfaction without overwhelming them [9]. It is also important to note that many games now are designed to combine features from multiple genres, challenging traditional classifications [10,11,12].

Initially perceived as a predominantly male form of entertainment, gaming has seen a shift towards a more balanced representation of sexes, with an increased number of women identifying as gamers [13]. Furthermore, gaming is no longer perceived as a child’s or adolescent’s activity, a view which has also changed as more and more adults take up video games as a pastime. Studies indicate that 61% of U.S. adults are playing video games, with the average age of a gamers being 36 [2]. This is attributable to the fact that teenage gamers grow into video game-playing adults. Early adulthood, defined as the period between 20 to 40 years of age, is described as a period of cognitive stabilization and peak performance, making this age range critical for research in terms of the effects of video games [14,15].

### 1.1. The Effects of Video Games on Cognitive and Affective Elements

The increased popularity and immersive nature of video games have made them a significant topic in neuropsychological research. However, findings are often inconclusive [16,17,18] and are often mediated by the game genre in question [19,20], independent from the platforms used [21].

A particular focus lies on the potential negative effects of violent video games on aggression, antisocial and externalized behavior, prosocial behavior, empathy, and desensitization to violence [22,23,24,25,26], with these effects being more prevalent in men [27]. However, this perspective has been challenged by studies arguing that the deleterious effects of violent video games are overstated, and result from less robust methodologies [28,29,30,31,32,33]. Additionally, some studies suggest that certain types of video games, such as RPGs, social games, and cooperative games can enhance empathy [34,35,36,37], whereas cooperative violent video games mitigate the empathy-reducing effects of violent content [38,39,40,41,42,43,44,45,46,47]. There is an interesting finding regarding the age of the players and empathy, which appears to impact adolescents more than adults or older adults [48]. This also raises questions about the onset of video game play, as there is evidence to suggest that the age one begins playing video games has a greater impact on cognitive abilities than the frequency of play, which could be attributed to greater brain plasticity during earlier years [49]. Finally, it should be noted that there are special video games being developed specifically for training empathy [50].

There has also been extensive research on the effects of video games on cognitive domains, including their potential for rehabilitating cognitive deficits. These cognitive domains commonly engaged during playing include visuospatial skills, short-term and working memory, attention, and psychomotor speed, which is also reported as hand–eye coordination

Most studies indicate that video games have positive effects on the visuospatial skills of players [51,52,53,54,55]. Action video games, in particular, require players to track and identify multiple moving objects simultaneously, potentially enhancing visuospatial skills. This genre, along with FPS games, have shown improvements in visuospatial short-term and working memory. However, some studies suggest that video games do not affect visuospatial memory, underscoring a need for further investigation [56]. Evidence also suggests a “dose-related” effect, with more video game play leading to greater improvements in visuospatial skills [57].

Attention is another domain frequently investigated in the context of video games. Video games offer an interactive experience that often demands constant player attention and engagement, depending on the specific type of video game [58,59,60]. Action video games, in particular, seems to benefit attention, with a plethora of studies demonstrating a positive relationship [61,62,63,64,65,66,67]. Action video games exhibit an interesting, sex-related effect on attention, with women benefiting more from playing such games than men [68]. Action games are not the only genre that improves the attention of their players; RPGs appear to exhibit the same level of beneficial effects on player’s attention as action video games [69]. Furthermore, the advent of virtual reality (VR) games significantly improve visuospatial skills compared to conventional ones [70]. However, some studies report no interaction between action games and attention [71,72], highlighting the need for more research.

Video games not only require attention and visuospatial skills, but they also demand fast player reactions to on-screen stimuli and psychomotor speed (hand–eye coordination). Both of these skills are reflected in the player’s reaction time, an important metric, both for gamers and researchers alike. Research suggests that video games can improve psychomotor speed and reaction times, with FPS games exhibiting particular promise in the case of psychomotor speed [73,74,75,76,77,78,79].

Verbal memory, both short-term and working, has not been extensively studied, offering inconclusive results. Some research supports the notion that video games provide benefits for one’s verbal memory [54,80], while other studies indicate either a negative relation, with games being detrimental to one’s verbal memory [81], or no relation at all [82]. Further investigation is needed to examine the effects of video games on verbal skills.

Another significant area of research involves the impact of video games on cognitive functions and their potential use in neuropsychological rehabilitation to battle cognitive decline [83]. Various studies have explored using video games as a way to help older adults reduce cognitive decline and retain cognitive levels [84,85,86,87,88]. Furthermore, there have been attempts to use video games as training devices to bolster cognitive abilities in older people, including memory [89,90,91,92]. Real-Time Strategy games (RTS) and Puzzle games have been identified to improve both short-term and working memory in older adults [93,94]. Interestingly, older adults seem to benefit cognitively more than younger ones from playing video games [95]. The general consensus is that more research is needed [96,97].

While numerous studies advocate for the cognitive and affective benefits of video game play, there is also substantial literature indicating no causal relationship between increased video game play and enhanced cognitive abilities [98,99]. As such, researchers must acknowledge that the scientific consensus on this relationship remains unsettled, with debates ongoing [18,98,100].

### 1.2. Aims of This Study

This study aims to contribute to the growing and mixed-result literature regarding the effects of video games on various cognitive and affective domains. Specifically, the Gaming Skill Questionnaire (GSQ) was used to assess the skills of adults in six different video game genres as well as in gaming in general. The effects of these skills were then assessed on six cognitive and one affective domain. Cognitive domains assessments included: verbal short-term memory and verbal working memory, using the Digit Span Test; visuospatial short-term memory and visuospatial working memory, using the Corsi Block Test; and psychomotor skills and attentional processing speed, using the Deary–Liewald Simple and Choice Reaction Time Tests. The affective domain was investigated for empathy using the EQ Questionnaire.

Our research hypothesis posits that experience with video games will significantly affect the cognitive skills investigated, as well as empathy, either positively or negatively. Furthermore, this study aims to elucidate whether these forms of digital engagement can predict performance in cognitive assessments and empathy measures to better understand the potential benefits and drawbacks of video game engagement. By integrating these findings, this research hopes to inform future studies and practical applications in cognitive training and mental health interventions.

## 2. Materials and Methods

### 2.1. Participants

The present study’s sample consisted of 88 adults, aged 20 to 40; 45 of which were female and 43 were male. Apart from age and sex, demographic data also included the education level of the participants, measured in years, ranging from 12 to 25 years. Their recruitment was conducted using the participants’ pools, email lists, and the social medias of the University of Edinburgh and the American College of Greece. Participation was voluntary and each participant signed a consent form before taking part in the study. Inclusion criteria required the absence of neurological and psychiatric diseases/disorders, any form of addiction, drug/alcohol abuse, and learning difficulties.

### 2.2. Materials

Demographic data for this study were collected using a custom questionnaire that incorporated questions relating to the participants’ age, sex, years of education, and exclusion criteria as described above.

#### 2.2.1. Gaming Skill Questionnaire (GSQ)

This questionnaire was used to quantify the skills of the participants in six different gaming genres as well as their overall gaming skills [101]. The GSQ included sections for each of the six gaming genre: sports games, first person shooter (FPS) games, role playing games (RPG), action–adventure games, strategy games, and puzzle games. Each section was comprised of two questions: one on the frequency of play, ranging from 1 (Never) to 6 (Everyday), and one on the self-perceived expertise in said genre, ranging from 1 (No skill) to 6 (Expert). Scoring on each genre’s gaming skills consisted of the sum of the scores from the two questions. Total Gaming Skill score was the sum of the gaming skills for each of the six sections. Thus, the test provided seven scores, sports games skill (SpGS), FPS games skill (FPSGS), role-playing games skill (RPGS), action–adventure games skill (AGS), strategy games skill (StGS), puzzle games skill (PGS), and total gaming skill (TGS) [101] (see Supplementary Material).

The GSQ has high reliability and validity, with a high Cronbach’s alpha (varying from 0.8 to 0.91 in the different sections), strong convergent validity (with item loadings at 0.69 at minimum and 1 at maximum), and excellent divergent validity (with low associations between the sections, ranging from 0.092 to just 0.002), thus justifying its inclusion in the present experiment [101]. The GSQ (English version) can be accessed here: http://dx.doi.org/10.13140/RG.2.2.27257.24160 (accessed on 20 August 2024).

#### 2.2.2. Digit Span Test (DST)

The Digit Span Test measures verbal memory by presenting sequences of digits for participants to verbally recall [102]. The sequences of digits progressively increase in length. There are two iterations of the DST: DST Forward and DST Backward. In DST Forward, the participants recall the sequences of digits in the same order that they were presented, while in the DST Backward, the participant has to recall them in the inverse order. Performance is calculated based on the longest correctly recalled sequence and the number of correct trials. DST Forward is specifically used to assess one’s verbal short-term memory, while DST Backward is used to assess one’s verbal working memory [103]. This difference warranted the inclusion of both iterations of DST in this experiment.

#### 2.2.3. Corsi Block Test (CBT)

The Corsi Block Test assesses visuospatial memory [104]. Participants are shown blocks on the screen, which sequentially light up, and must recall the sequence. The sequence of blocks lighting up progressively increases in length. There are two iterations: CBT Forward and CBT Backward. In CBT Forward, the participants have to recall the sequence in the same order, while in CBT Backward, the participant has to recall the sequence in the inverse order [105]. Performance is calculated based on the longest sequence successfully recalled and the number of correct trials. CBT Forward assesses visuospatial short-term memory, while CBT Backward assesses visuospatial working memory [106,107,108].

#### 2.2.4. Deary–Liewald Reaction Time Task (DLRTT)

This task is used to assess the reaction time and the information processing speed of the participants. There were two iterations of DLRTT implemented in the present study: The Deary–Liewald Simple Reaction Time Task (DLSRTT), where participants press a key as fast as possible when a visual stimulus (a cross is presented) [109], and the Deary–Liewald Choice Reaction Time Task (DLCRTT), where participants press one of two buttons, depending on whether a left or right arrow appears on the screen [109]. Reaction time measured in milliseconds is the score for each task. DLSRTT measures psychomotor skill, while DLCRTT measures attentional speed [109].

#### 2.2.5. Empathy Quotient Questionnaire (EQ)

The EQ questionnaire is a tool designed to evaluate the empathy of adults. It includes 60 questions, 40 of which are designed to assess the perception and influence of the emotions of others and 20 are filler items [110]. The participant is presented with each question and has to choose the best-suited reply from four available, ranging from totally agree, somewhat agree, somewhat disagree, and totally disagree. It is designed to be completed within 5 to 10 min, with scores ranging from 0 to 80 [111]. The questionnaire appears to be both highly reliable and valid, which holds true also for the Greek version of the EQ Questionnaire that was used, with a Cronbach’s alpha of 0.902, an Intraclass Correlation Coefficient of 0.928, and a good fit in the Confirmatory Factor Analysis [112].

### 2.3. Procedure

The study was conducted in a controlled laboratory setting. Each participant was alone with the researcher during data collection. Participants received a detailed briefing on the procedure, tests, type of data collected, and the confidentiality as well as the adherence to GDPR laws. Informed consent was obtained prior to participation.

Initially, participants had to fill in the demographic questionnaire, followed by the completion of the GSQ. Extra care was taken to ensure that the participants provided accurate information.

Subsequently, the three tasks were administered, namely DST, CBT, and DLRTT, as they were described in the Materials section. In each type of test, the sequence was maintained as proposed in the literature, with the Forward iteration being administered first, followed by the Backward one, in the cases of DST and CBT, while for DLRTT, the Simple iteration was administered first, followed by the Choice one [102,105,109]. The order of the three tests was randomized using a Latin square design to avoid primacy effects.

After the administration of all three tests, the participants completed the EQ questionnaire, striving to capture thoughtful and accurate answers, they were reminded of the importance of truthfully replying. Finally, the participants were debriefed, reminded of their rights regarding their participation in the study and their personal data.

### 2.4. Statistical Analysis

Statistical analyses for this study were performed using the R programming language (version 4.3.3) [113] within the RStudio environment (version AGPL v3) [114]. Essential R packages were utilized, including psych package version 2.4.6.26. [115] for correlation, regression, ANOVA, and post hoc comparison analyses, and ggplot2 package version 3.5.1. [116] for generating visual plots. The analysis began with descriptive statistics to provide a comprehensive overview of the sample demographics and test scores. Pearson’s correlations were performed to identify potential correlations between variables.

#### 2.4.1. Regression Analysis Process

Linear Simple regressions were performed with the goal of uncovering the predictive values of all individual predictor variables on the criterion variables. For the Linear Multiple Regression models, the variables considered included demographic data, gaming skill levels, the cognitive test scores, and the EQ questionnaire score. The incremental approach was chosen, which consisted of initial Single-Predictor models, one for each predictor, being created. These allowed the identification of the most effective variable. These were followed by Dyadic Predictor Models, including two predictors, with the inclusion of the most effective variables from the Single-Predictor Models. The performances of Dyadic Predictor Models and of the Single-Predictor Models were compared to identify the most effective combinations. Finally, an Incremental Model Development process was conducted, whereby predictors were added in each phase, with the resulting model being compared to the previous one. This leads to progressively more complex models, with the process being halted when the addition of a new predictor led to no significant improvement in the model’s performance. The optimal model was chosen as the simplest possible model with superior performance compared to more complex ones. This allowed the selection of a robust and accurate representation of the most influential variables in the chosen models.

The regression analysis commenced with verifying data normality using the Shapiro–Wilk Normality test (shapiro.test function from the stats package [113]) and confirming homoscedasticity with the Non-Constant Error Variance test (ncvTest function from the car package [117]). Multicollinearity was assessed by calculating the variance inflation factor (VIF) for each predictor within the models using the vif function from the car package [117]. Linear regression analyses were employed to explore various predictors of cognitive functions and behaviors, utilizing the lm function from the stats package [113]. Models were compared using the Akaike Information Criterion (AIC), Bayesian Information Criterion (BIC), F statistic significance level, and the proportion of explained variance (R^2^). For the multiple linear regression analyses, variables considered included demographic factors, gaming skill levels, cognitive test scores, and the EQ questionnaire score. An incremental approach was adopted for the analytical process:

1. Single-Predictor Models: Initially, separate models were developed for each predictor to identify the most effective variable based on performance.
2. Dyadic Predictor Models: Subsequently, models incorporating two predictors were constructed, consistently including the most effective variable from the single-predictor models. The performance of these dyadic models was then evaluated and compared to the Single-Predictor models to ascertain the most effective combination.
3. Incremental Model Development: This iterative approach involved adding a predictor in each phase and comparing the performance of increasingly complex models. The process continued until the inclusion of new variables no longer significantly improved the models’ performance. The optimal model was determined when a simpler model from an earlier phase demonstrated superior performance compared to a more complex model from a later phase. This ensured the final model was robust and accurately reflected the most influential variables identified in the study.

#### 2.4.2. One-Way ANOVA Analyses

One-way ANOVA analyses were performed on the data set using the aov function from the stats package [113], with participants divided into Low, Medium, and High Gaming Skill Levels based on their GSQ scores. This division was used to examine differences in cognitive performance on the three tests and empathy across different levels of gaming skills in individual genres and overall gaming proficiency. Post-hoc Bonferroni corrected comparisons were conducted using the pairwise.t.test function to uncover specific differences between gaming skill levels. This comprehensive approach ensured a detailed understanding of how different gaming skill levels impact cognitive and affective functions.

## 3. Results

The sample (*N* = 88) included 45 female and 43 male adults, aged 20 to 40. Table 1 presents the descriptive statistics, which include the demographics, namely age and years of education, their gaming skills in all six genres inquired about in the GSQ, as well as their total gaming skill. Furthermore, it also includes the descriptive statistics of the three cognitive tests, specifically the DST Forward and Backward, the CBT Forward and Backward, and the DLRTT Simple and Choice, as well as for the EQ questionnaire. Table 1 includes the total values but also the values based on the gaming skills of the participants, when divided into Low, Medium, and High based on their Gaming Skills Score.

Comparing the three levels of gaming skill, namely Low, Medium, and High, it is evident that higher gaming skill individuals tend to be older, with the mean age of the participants increasing along with the increase in gaming skill. Regarding education level, those with medium gaming skills exhibited the highest, followed by those with low and finally those with high gaming skills. In all genres of games, specific skills increased together with the general gaming skill level increase. In both Forward and Backward iterations in both DST and CBT, the higher the gaming skill level, the better the score. In DLSRT, the reaction time was the lowest in the high gaming level group but worst in the medium gaming level group. In DLCRT, the higher the gaming level, the lower the reaction time. Finally, in the EQ, the higher the gaming level, the lower the EQ score.

### 3.1. Correlations

Pearson’s correlations are reported in Table 2. Starting with the demographic factors, age seems to be significantly but weakly positively correlated with the scores in both CBT forward and backward as well as significantly weakly negatively correlated with the EQ score. Education on the other hand, resulted in no significant correlations with any of the seven tests.

Regarding gaming skills, sports gaming was significantly weakly positively correlated only with the DST Backward. FPS gaming skill was significantly weakly positively correlated with both CBT forward and backward, and significantly weakly negatively correlated with both Simple and Choice iterations of the DLRTT. RPGS too was significantly weakly positively correlated with both Forward and Backward iterations of the CBT, but also significantly weakly negatively correlated with the scores of the EQ Questionnaire. AGS was significantly weakly positively correlated with the scores of DLSRT and DLCRT tasks. StGS only resulted in a significantly weak positive correlation with the Forward variant of the CBT. PGS was significantly weakly positively correlated only with the two iterations of the CBT, forward and backward. Finally, TGS was significantly weakly positively correlated with almost all of the tests, specifically DST Forward, CBT Forward and Backward, DLSRT, and DLCRT, with only the Forward variant of DST and the EQ Questionnaire not achieving significance.

### 3.2. Regressions

Table 3 presents the optimal regression models for each of the seven tests conducted. For DST-F, a null model was identified as the best, indicating no predictive factors for DST-F performance. In contrast, the best regression model for DST-B achieved an R^2^ of 0.12, demonstrating a weak relationship between verbal working memory and the sole predictor, RPGS. Specifically, RPGS showed a beta coefficient of 0.35, suggesting that increased RPGS is moderately associated with improved verbal working memory. The CBT-F model had an R^2^ of 0.20, indicating a moderate relationship between visuospatial short-term memory and the predictors: age and RPGS. RPGS was the stronger predictor, with a beta coefficient of 0.32, signifying that higher RPGS is moderately linked to better performance in visuospatial short-term memory. Age had a beta coefficient of 0.24, implying that older participants achieved moderately better results in CBT-F. For CBT-B, the best model yielded an R^2^ of 0.19, indicating a moderate relationship between visuospatial working memory and the predictors: age and PGS. PGS was the strongest predictor with a beta coefficient of 0.25, indicating that higher PGS is moderately associated with better performance in CBT-B. Age was also positively correlated with visuospatial working memory, with a beta coefficient of 0.23, suggesting that older individuals performed moderately better.

The DLSRT model exhibited an R^2^ of 0.16, indicating a moderate relationship between psychomotor speed and AGS, the sole predictor. AGS had a beta coefficient of −0.39. Since lower scores on the Deary–Liewald test indicate better performance, an increase in AGS is moderately correlated with faster psychomotor response times. Similarly, the best model for DLCRT had AGS as the sole predictor, with an R^2^ of 0.10, suggesting a weak relationship between DLCRT and AGS. The beta coefficient of −0.32 indicated that higher AGS is moderately associated with faster attention speed. The best regression model for EQ yielded an R^2^ of 0.07, suggesting a weak relationship between empathy and RPGS. The beta coefficient for RPGS was −0.26, indicating that higher RPGS is moderately associated with lower empathy. Overall, while the regression models were successfully generated for six of the seven dependent variables, with gaming skills serving as predictors, the R^2^ values varied from moderate (0.20 for CBT-F) to small (0.07 for EQ). This suggests that while the relationships are significant, their impact ranges from moderate to weak depending on the cognitive domain [118].

### 3.3. ANOVA

The ANOVA analyses were performed to examine the differences among the levels of gaming skills in terms of cognitive performance in six different domains and empathy. For this reason, participants were divided into three groups, based on their gaming skills. The low group included 30 participants, the middle group and the high group included 29 participants each. The descriptive statistics are presented in Table 1.

**Figure 1:**
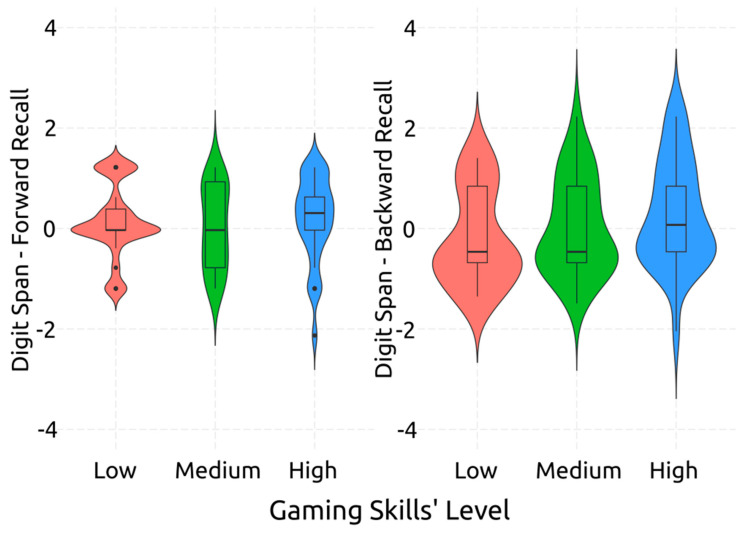
Post-Hoc Comparisons: Performance on Digit Span Tasks per Gaming Level.

The ANOVA uncovered no significant small sized effect of gaming skill level on the verbal short-term memory of the participants [*F*(2,85) = 0.69, *p* = 0.507, *η*^2^*_p_* = 0.02], as well as on their verbal working memory [*F*(2,85) = 2.04, *p* = 0.136, *η*^2^*_p_* = 0.05], as they were measured by DST Forward and Backward, respectively. These findings are visualized in Figure 1, and they indicate that gaming skill level is not significantly associated with either the verbal short-term memory or the verbal working memory of adults.

**Figure 2:**
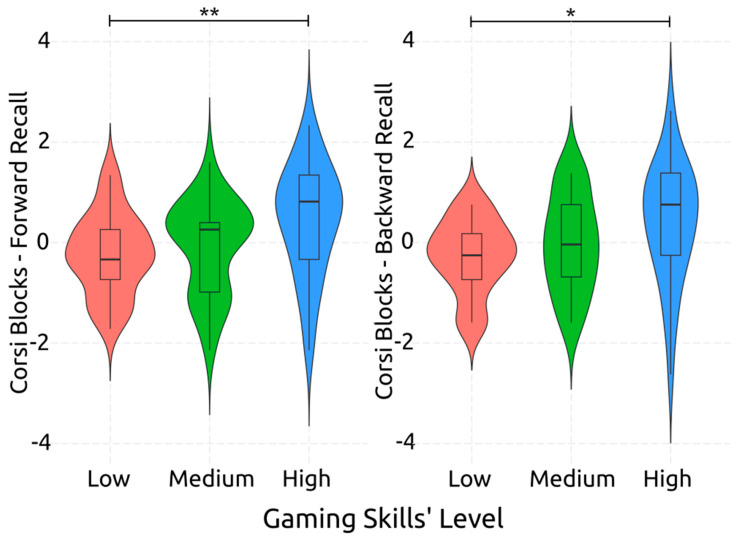
Post-Hoc Comparisons: Performance on Corsi Block Tasks per Gaming Level. The asterisks denote statistically significant differences between gaming skill levels. The double asterisk (**) indicates *p* < 0.01, while the single asterisk (*) indicates *p* < 0.05, highlighting the significant differences between low and high gaming skill groups in both forward and backward recall tasks.

Conversely, visuospatial memory performance was significantly related to gaming skill level. Specifically for visuospatial short-term memory, as measured by CBT-Forward, the effect of gaming skill level was significant and medium-sized [*F*(2,85) = 4.99, *p* = 0.009, *η*^2^*_p_* = 0.10]. Visuospatial working memory, measured by CBT Backward, was associated accordingly with gaming skill level, with a significant medium-sized effect [*F*(2,85) = 4.08, *p* = 0.020, *η*^2^*_p_* = 0.09]. The post-hoc comparisons, which are visually presented in Figure 2, reveal that there is a medium effect size when comparing high gaming skill level individuals to low gaming skill level ones, with the former having substantially better visuospatial short-term memory than the latter ones [*d* = 0.78, *p* = 0.009]. The same finding was uncovered in the case of visuospatial working memory [*d* = 0.73, *p* = 0.017]. Of interest was the fact that there was a marginally insignificant medium-sized effect between medium and high gaming skill levels on the performance in the visuospatial short-term memory task [*d* = 0.57, *p* = 0.070].

**Figure 3:**
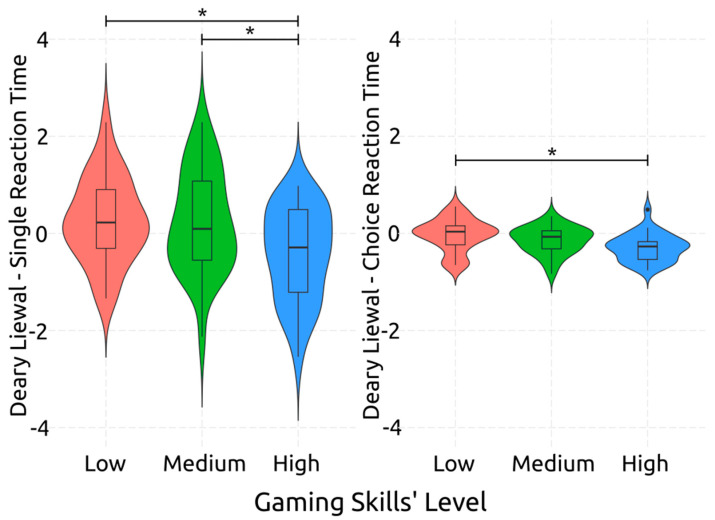
Post-Hoc Comparisons: Performance on Deary–Liewald Reaction Time Tasks per Gaming Level. The asterisks denote statistically significant differences between gaming skill levels. The single asterisk (*) indicates *p* < 0.05, highlighting significant differences between high and medium skill levels, as well as between high and low skill levels, in single reaction time. Additionally, * indicates *p* < 0.05, showing significant differences between low and high skill levels in choice reaction time.

Psychomotor skills also seem to be significantly related to the gaming skills levels of individuals. Specifically for motor speed, measured by DLSRTT, the effect of gaming skills level was significant and medium sized [*F*(2,85) = 4.36, *p* = 0.016, *η*^2^*_p_* = 0.09], and the same held true for the case of attentional speed, as measured by DLCRTT [*F*(2,85) = 3.10, *p* = 0.049, *η*^2^*_p_* = 0.07]. The post-hoc comparisons, visualized in Figure 3, unveil that, for the case of motor speed, individuals with high gaming skills are faster than those with medium gaming skills, showing a moderately sized significant effect [*d* = 0.62, *p* = 0.048] as well as than those with low gaming skills, showing an effect that is also significant and moderately sized [*d* = 0.73, *p* = 0.028]. Furthermore, the attentional processing speed of the participants with high gaming skill levels was faster than those with low gaming skill levels, this effect also being significant and medium sized [*d* = 0.47, *p* = 0.047].

**Figure 4:**
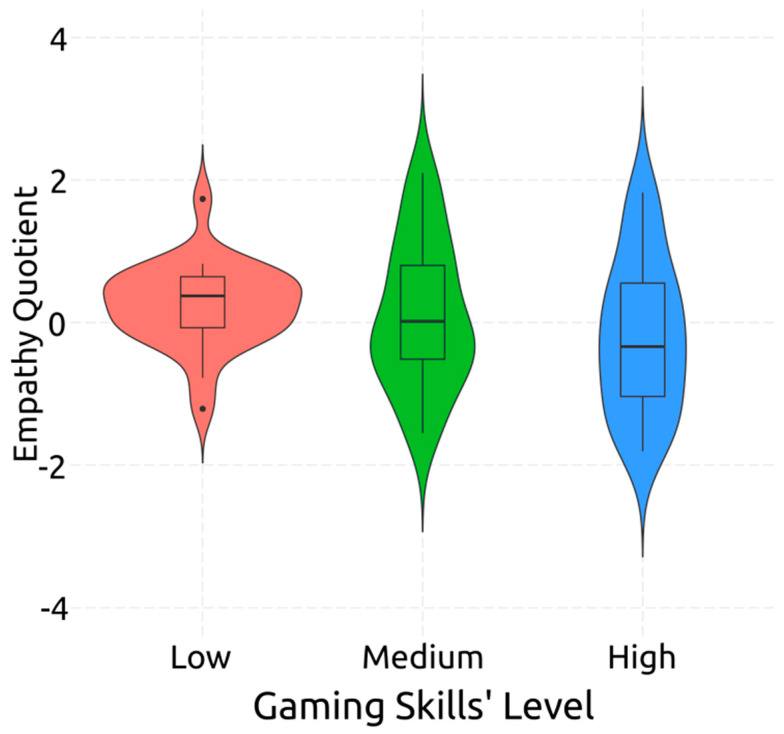
Post-Hoc Comparisons: Performance on Empathy Quotient Questionnaire per Gaming Level.

Finally, regarding Empathy, ANOVA analysis did not uncover any significant effect of gaming skill level on it [*F*(2,85) = 0.92, *p* = 0.404, *η*^2^*_p_* = 0.02]. It has to be noted that there was a decreasing trend, inversely related to gaming skill level, hinted at in Figure 4, but not reaching significance.

## 4. Discussion

This study aimed to investigate the impact of video game engagement on cognitive functions and empathy. Specifically, it focused on investigating the effects of gaming skills across six different gaming genres and gaming generally on six cognitive abilities and on empathy. Key findings from this study indicate that high gaming skills were consistently associated with better visuospatial memory, both short-term and working, and faster psychomotor and attentional speed. Especially for psychomotor speed, high gaming skill resulted in faster response times than both low and medium gaming skill levels.

### 4.1. Gaming Skill Level, Cognitive Functions, and Empathy

#### 4.1.1. Verbal Short-Term Memory

Verbal short-term memory, as measured by the DST-F, was a variable in this study. The non-significant ANOVA results suggest that verbal short-term memory is not influenced by gaming skill levels, and the null result from the regression analysis further reinforces this conclusion. These findings collectively indicate that none of the predictive factors, such as gaming proficiency or demographic characteristics, were associated with verbal short-term memory in the sample studied.

These findings align with the broader debate in the literature regarding the impact of video game play on verbal short-term memory. Some studies have posited that video game play, particularly in action or strategy-based genres, may offer cognitive benefits by enhancing memory retention through rapid decision-making and information processing demands [54,80]. These findings have led to the hypothesis that certain gaming experiences could improve short-term memory in players, especially those regularly engaging with cognitively demanding games.

Conversely, other research has suggested that video game play may exert a negative influence on verbal short-term memory. These detrimental effects are often attributed to the cognitive load imposed by complex gaming environments, which may divert attention away from verbal memory tasks or lead to cognitive fatigue [81]. In contrast, several studies have failed to identify any significant relationship between video game play and verbal short-term memory, arguing that gaming may not affect this cognitive domain at all [82]. These mixed results highlight the complexity of this issue and underscore the need for further investigation.

The absence of significant findings in both the ANOVA and regression analyses in our study reflects the ambiguity found in the literature. It is possible that the relationship between video game play and verbal short-term memory is more nuanced than previously thought, with factors such as game genre, individual differences in cognitive ability, age onset of video game player, and the frequency of play potentially moderating the effects. Future research should explore these variables in greater depth, possibly employing longitudinal designs to assess how sustained video game play influences verbal short-term memory over time. While our results suggest gaming skill levels do not predict verbal short-term memory, further investigation, focusing on game-specific factors and cognitive profiles, is needed to resolve this debate.

#### 4.1.2. Verbal Working Memory

The ANOVA for DST-B revealed no significant differences across gaming skill levels (low, medium, and high), similar to the findings for DST-F, reflecting the mixed evidence in the literature on the effects of video games on verbal working memory [54,80,81,82]. These results suggest that, at least in this sample, gaming skill levels do not significantly influence verbal working memory as measured by DST-B.

However, in contrast to DST-F, the best regression model for DST-B identified RPGS as a significant predictor. This aligns with previous research that has suggested a positive relationship between video game play, particularly RPGs, and improvements in verbal working memory due to the cognitive demands of complex storylines and strategic planning [54,69]. Additionally, significant correlations were observed between DST-B scores and SpGS, RPGS, and TGS, further indicating potential links between these specific gaming genres and verbal working memory. These findings add to the growing body of research that highlights genre-specific cognitive benefits of video games, particularly for RPGs and strategy-based games. However, it is important to note that while these correlations suggest possible connections, they do not imply causality, and further research is needed to explore these relationships more thoroughly.

#### 4.1.3. Visuospatial Short-Term Memory

This study measured visuospatial short-term memory using CBT-F. The ANOVA analysis revealed a significant difference between high and low gaming skill levels, with high-skill gamers performing better. This outcome aligns with existing literature, which consistently suggests that video game play, especially those requiring spatial navigation or processing spatial information, enhance visuospatial short-term memory [80,93,119,120,121]. The best regression model further identified age and RPGS as significant predictors, reinforcing the role of RPGs in improving visuospatial skills [69,122].

An inverse relationship between age and visuospatial short-term memory was also identified, consistent with previous research indicating a decline in visuospatial abilities after the mid-20s [85,123]. This decline could be attributed to age-related changes in cognitive processing speed and spatial awareness. In addition, CBT-F was significantly correlated with age, FPSGS, RPGS, StGS, and TGS, suggesting that a broad spectrum of video game genres, in combination with age, significantly relate to visuospatial short-term memory. These findings provide further evidence that a variety of gaming experiences—ranging from fast-paced first-person shooters to strategic role-playing games—are linked to enhancements in visuospatial cognitive functions.

#### 4.1.4. Visuospatial Working Memory

The ANOVA conducted on CBT-B revealed that participants with high-level gaming skills performed significantly better in visuospatial working memory compared to those with low-level gaming skills. This result is consistent with the majority of the literature, which suggests that regular video game play enhances visuospatial working memory [52,54,124,125,126,127]. The best regression model identified PGS and age as significant predictors. Puzzle games, in particular, positively associated with visuospatial working memory, likely due to their cognitive demands on spatial reasoning and problem-solving [69,94,122].

Interestingly, increasing age was also associated with improved visuospatial working memory, a finding that contrasts with much of the literature that suggests cognitive decline begins around the age of 24 [85,123]. This unexpected result may warrant further investigation to explore whether certain factors, such as cognitive engagement through gaming, can mitigate the effects of age-related cognitive decline. In addition, CBT-B was significantly correlated with Age, FPSGS, RPGS, PGS, and TGS, indicating that playing a variety of gaming genres, alongside gaming skills in general, is significantly associated with enhanced visuospatial working memory. The positive association between these gaming genres—FPS, RPGs, and puzzle games—and visuospatial working memory has been well-documented in prior studies, reinforcing the role of video game play in supporting spatial cognition [94,122].

#### 4.1.5. Psychomotor Speed

The DLSRTT was employed in this study to measure psychomotor speed. The ANOVA results for DLSRTT were notable, as individuals with high gaming skill levels demonstrated significantly better psychomotor speed compared to both medium- and low-skill participants. Given that psychomotor speed involves hand–eye coordination, this finding aligns with a substantial body of research suggesting that video game play enhances this cognitive domain [53,73,74,78,128]. The demands of fast-paced gaming, particularly in action-based genres, likely contribute to these improvements, as players must react quickly to visual stimuli and execute precise motor responses.

Moreover, the best regression model identified AGS as a significant predictor, further reinforcing the link between action games and enhanced psychomotor skills [78,129,130]. Action games, which require rapid decision-making and motor responses, are well-documented in their ability to improve psychomotor functions. The correlation analysis also revealed significant relationships between FPSGS, AGS, and TGS, supporting the findings from both the ANOVA and regression analyses. These correlations emphasize the role of various gaming genres, particularly action and first-person shooter games, in contributing to better psychomotor speed, underscoring the cognitive benefits associated with regular video game play.

#### 4.1.6. Attentional Speed

Attentional speed was measured using the DLCRT. The ANOVA results revealed that participants with high gaming skill levels had significantly faster attentional speed compared to those with lower skill levels, a finding that aligns with existing literature [63,73,130,131,132,133,134]. The cognitive demands of fast-paced video games, particularly in action genres, may explain these improvements in attentional processing speed, as these games require players to quickly identify, process, and respond to dynamic stimuli.

This finding is further supported by the best regression model for DLCRT, where AGS was identified as the sole predictor, reinforcing the positive association between action game skills and attentional speed. The role of action video games in enhancing attentional speed has been well-documented, with studies consistently highlighting their benefits for cognitive processing speed and attentional control [62,64,135,136,137]. Additionally, FPSGS, AGS, and TGS were significantly correlated with DLCRT, further validating the outcomes of both the ANOVA and regression analyses. These correlations underscore the contribution of multiple gaming genres, particularly action and first-person shooter games, to faster attentional processing.

#### 4.1.7. Empathy

The ANOVA for empathy, as measured by the EQ questionnaire, revealed no significant association between gaming skill levels and empathy, consistent with prior studies. [29,38,42,44]. However, it is important to note that there was a non-significant downward trend in empathy scores as gaming skill levels increased, suggesting a possible inverse relationship. This trend hints at the possibility that higher gaming skills might be associated with lower levels of empathy, aligning with a segment of the literature that suggests video games may have a negative effect on empathy [22,138,139,140,141,142].

The best regression model identified RPGS as the sole predictor, supporting the notion that increased gaming skill—reflecting both experience and frequency of play—may be associated with decreased empathy. This finding reinforces the part of the literature that highlights the potential negative effects of video games on empathy [22,138,139,140,141,142]. This contrasts with existing research that argue RPGs enhance empathy due to their narrative and character-driven gameplay [39,43,46,143] raising questions about how different genres impact social and emotional skills. Correlations with RPGs and age also suggested that older individuals, as well as those with higher RPGS scores, exhibited lower levels of empathy, adding nuance to the broader discussion of how gaming and age might interact with socio-emotional outcomes.

It is important to note, that while the best regression models for the various cognitive skills and empathy consistently included gaming skills as significant predictors, the amount of variance explained by these factors was relatively small to moderate. This observation is crucial for contextualizing the results, as it indicates that while gaming skills—across different genres—are associated with cognitive performance and empathy, these effects are modest. Thus, the significance of these associations should be interpreted with caution, emphasizing that video game play is only one of many factors influencing cognitive skills and emotional attributes such as empathy.

### 4.2. Diverse Video Game Genres and Cognition

#### 4.2.1. FPS & Action Games

FPS and action games demand quick reflexes, as success in these games is often determined by split-second reactions. In many cases, the outcome of firefights favors the player who reacts and shoots first, making every millisecond critical to success. Additionally, recognizing and identifying an opponent before engagement is key to effective performance. These requirements underscore the need for high levels of both reaction speed and attentional speed when playing FPS and action games [61,62,144,145]. The findings from the present study align with these observations, as evidenced by the significant associations between AGS and attentional speed, and the positive correlations between AGS and both attentional and psychomotor speed. Furthermore, the significant correlations between FPS skills and visuospatial short-term and working memory highlight the broader cognitive demands of FPS games, reinforcing the idea that these games enhance not only reflex-based skills but also higher-order cognitive functions.

#### 4.2.2. Puzzle Games

Puzzle games, particularly those utilizing 3D graphics, often present players with complex, spatially intricate puzzles that require a deep understanding of level structure and the application of basic physics principles. Games like Portal exemplify this genre, as players must engage in problem-solving that involves spatial reasoning and the manipulation of the environment to progress. The cognitive demands of such games help explain the positive association between PGS and visuospatial working memory observed in this study, a relationship also supported by previous research [94,146,147]. The intricate spatial tasks required by puzzle games likely promote the development of visuospatial working memory, as players are continuously challenged to understand and manipulate 3D environments to solve puzzles. This connection between puzzle games and visuospatial memory emphasizes the cognitive benefits offered by this genre, particularly in enhancing spatial reasoning and problem-solving skills.

### 4.3. Limitations and Future Studies

This study employed a pseudorandom sampling method with age restrictions, focusing on early adulthood, which may limit the generalizability of the findings to broader populations. While the sample size of 88 participants provided adequate statistical power, future research would benefit from larger and more diverse samples that capture a wider range of gaming habits, cognitive profiles, and age groups, such as middle-aged and older adults. The recruitment, limited to individuals from the University of Edinburgh and the American College of Greece, also restricted the cultural and socioeconomic diversity of the sample. Future studies should aim to include participants from more varied backgrounds to enhance the generalizability and applicability of the results.

The cross-sectional nature of the study offers valuable insights into the immediate cognitive and affective impacts of video game play but the long-term effects were not measured. Future studies should adopt longitudinal designs to investigate how sustained video game engagement affects cognitive functions, such as memory and attention, as well as socio-emotional domains like empathy, over time. These designs would also help address the potential for reverse causation, examining whether individuals with specific cognitive or affective traits are more likely to engage in certain genres of video games. Additionally, given the genre-specific effects observed in this study, future research may investigate how different video game genres influence cognitive and affective processes, applying these genres to cognitive training or therapeutic contexts.

Moreover, the tools used to assess empathy, such as the Empathy Quotient questionnaire, while valid, may not have been sensitive enough to capture subtle changes in empathy related to video game play. Future research should consider employing more refined measurement tools or advanced neurocognitive techniques to better assess shifts in empathy. Studies with experimental designs in controlled settings could further explore the potential of video games as tools for enhancing empathy or other cognitive skills, particularly in interventions aimed at improving mental health or social functioning. Overall, while the present study provides valuable insights, future research with more diverse samples, longitudinal approaches, and genre-specific analyses will be critical to deepening our understanding of the long-term cognitive and emotional impacts of video game play.

## 5. Conclusions

The present study aimed to investigate the effects of video game engagement, a globally popular form of entertainment, on various cognitive domains and empathy. The findings revealed that some cognitive functions, including verbal short-term memory and verbal working memory, were not associated with different levels of gaming skills. However, other cognitive tasks—specifically visuospatial short-term and working memory, psychomotor speed, and attentional speed—were significantly enhanced in individuals with high gaming skills compared to those with lower gaming abilities. Notably, high-level gamers demonstrated superior psychomotor speed compared to both low- and medium-skill participants.

The study also highlighted genre-specific effects, indicating that different video game genres are associated with distinct cognitive benefits. Role-playing games (RPGs) were positively linked to improvements in verbal working memory and visuospatial short-term memory, while they were negatively correlated with empathy. Action–adventure games contributed to better psychomotor speed, reflecting enhanced hand–eye coordination, as well as faster attentional speed. Puzzle games, on the other hand, were associated with improvements in visuospatial working memory.

These findings contribute to the growing body of literature on the cognitive effects of video game play, emphasizing the varied influence of different video game genres on specific cognitive functions. The results underscore the importance of considering genre when examining the cognitive impacts of video games, as different types of games appear to offer unique cognitive benefits. Nevertheless, further research is necessary to deepen our understanding of the long-term effects of video game engagement and to explore the potential for video games to be used in cognitive training or therapeutic interventions.

## Figures and Tables

**Table 1:** Descriptive Statistics of Demographics, Gaming Skills per Genre and Total, Cognitive Performance, and Empathy per Gaming Skill Level and Overall.

	Gaming Skill	Mean	SD	Range
Age	Low	26.93	4.274	20–38
	Medium	29.00	5.359	21–40
	High	29.28	4.122	21–38
	Total	28.39	4.680	20–40
Education	Low	16.40	2.343	12–21
	Medium	17.24	3.055	12–25
	High	15.93	2.298	12–20
	Total	16.52	2.610	12–25
Sport Games Skill	Low	2.27	0.450	2–3
	Medium	3.17	0.928	2–6
	High	3.69	1.228	2–6
	Total	3.03	1.090	2–6
FPS Games Skill	Low	2.13	0.346	2–3
	Medium	2.72	0.797	2–5
	High	4.41	1.615	2–9
	Total	3.08	1.420	2–9
RPG Games Skill	Low	2.10	0.305	2–3
	Medium	3.03	1.239	2–7
	High	6.86	2.656	2–11
	Total	3.98	2.660	2–11
Action Games Skill	Low	2.27	0.640	2–4
	Medium	3.62	1.898	2–10
	High	6.31	2.140	2–11
	Total	4.05	2.370	2–11
Strategy Games Skill	Low	2.07	0.365	2–4
	Medium	2.76	0.988	2–5
	High	4.86	2.532	2–12
	Total	3.22	1.960	2–12
Puzzle Games Skill	Low	2.43	0.626	2–4
	Medium	3.52	1.326	2–6
	High	5.52	2.309	2–11
	Total	3.81	2.02	2–11
Total Gaming Skill	Low	13.27	1.143	12–15
	Medium	18.83	2.633	16–24
	High	31.66	5.627	25–43
	Total	21.16	8.540	12–43
Digit Span Forward Recall	Low	16.13	2.849	8–20
	Medium	16.07	2.685	9–20
	High	16.79	2.411	11–20
	Total	16.33	2.650	8–20
Digit Span Backward Recall	Low	13.97	3.429	9–19
	Medium	15.00	3.082	8–20
	High	15.69	3.486	3–20
	Total	14.88	3.380	3–20
Corsi Block Forward Recall	Low	7.23	2.687	3–13
	Medium	7.86	3.020	2–14
	High	9.90	3.745	2–17
	Total	8.32	3.340	2–17
Corsi Block Backward Recall	Low	6.97	2.632	3–11
	Medium	7.90	3.457	3–13
	High	9.55	3.709	2–16
	Total	8.13	3.430	2–16
Deary-Liewald Single Reaction Time	Low	282.60	46.154	231–462
	Medium	286.07	57.681	207–462
	High	255.59	27.800	195–303
	Total	274.84	47.070	195–462
Deary-Liewald Choice Reaction Time	Low	470.03	119.097	331–804
	Medium	433.55	69.300	296–641
	High	395.21	48.565	310–521
	Total	433.35	89.340	296–804
Empathy Quotient	Low	44.57	9.134	18–62
	Medium	42.86	12.397	18–66
	High	40.55	12.126	22–63
	Total	42.68	11.280	18–66

FPS = First-Person Shooting; RPG = Role Playing Games.

**Table 2:** Pearson’s r Correlations: Cognitive Performance, Empathy, Demographics, and Gaming Skills.

	DST-FR	DST-BR	CBT-FR	CBT-BR	DLSRT	DLCRT	EQ
Age	0.08	0.20	0.30 **	−0.27 *	0.14	0.13	−0.24 *
Education	−0.06	−0.03	−0.18	−0.14	0.09	0.16	−0.07
SpGS	0.04	0.25 *	0.13	0.18	−0.17	−0.18	−0.08
FPSGS	−0.02	0.11	0.25 *	0.23 *	−0.27 *	−0.23 *	−0.21
RPGS	0.17	0.35 *	0.35 *	0.27 *	−0.10	−0.16	−0.26 *
AGS	0.07	0.13	0.10	0.12	−0.39 ***	−0.32 **	−0.12
StGS	0.03	−0.01	0.34 *	0.18	−0.12	−0.03	−0.21
PGS	0.20	0.10	0.25 *	0.28 **	−0.18	−0.21	−0.08
TGS	0.13	0.22 *	0.29 **	0.27 *	−0.26 *	−0.24 *	−0.20

DST = Digit Span Test; CBT = Corsi Blocks Test; FR = Forward Recall; BR = Backward Recall; DL = Deary-Liewald; SRT = Single Reaction Time; CRT = Choice Reaction Time; EQ = Empathy Quotient; SpGS = Sport Games Skill; FPSGS = First-Person Shooting Games Skill; RPGS = Role Playing Games Skill; AGS = Action-Adventure Games Skill; StGS = Strategy Games Skill; PGS = Puzzle Games Skill; TGS = Total Gaming Skills. * *p* ≤ 0.05, ** *p* ≤ 0.01, *** *p* ≤ 0.001.

**Table 3:** Best Regression Models for Predicting Cognitive Performance and Empathy.

Predicted	Predictors	β Coefficient	*p*-Value (β)	R^2^
Digit Span Forward Recall	Null Model	-	-	-
Digit Span Backward Recall	Role-Playing Games Skills	0.35	0.004 *	0.12
Corsi Blocks Forward Recall	Age	0.24	0.022 *	0.20
	Role-Playing Games Skills	0.32	0.003 **	
Corsi Blocks Backward Recall	Age	0.23	0.028 *	0.19
	Puzzle Games Skills	0.25	0.018 *	
Deary–Liewald Single Reaction Time	Action Games Skills	−0.39	0.001 ***	0.16
Deary–Liewald Choice Reaction Time	Action Games Skills	−0.32	0.008 **	0.10
Empathy Quotient	Role-Playing Games Skills	−0.26	0.014 *	0.07

* *p* ≤ 0.05, ** *p* ≤ 0.01, *** *p* ≤ 0.001.

## Data Availability

The data presented in this study are available on request from the corresponding author. The data are not publicly available due to ethical approval requirements.

## Supplementary Materials

The following supporting information can be downloaded at: http://dx.doi.org/10.13140/RG.2.2.27257.24160, accessed on 10 January 2024.
